# Westminster Hospital

**Published:** 1829-01-01

**Authors:** 


### XLVI.

### WESTMINSTER HOSPITAL.

This ancient hospital has received, by will of the late Mr. Tillard, of Canterbury, the sum of 10,000 pounds in aid of its Building Fund, which has been languishing since the year 1826. It is to be hoped the new hospital will soon be commenced, and upon that scale which will render it worthy of the reputation the old one has, for more than a century, enjoyed. The present building accommodates only 92 patients, but the number of applicants is so great, that it is distressing to see how many are weekly deferred for want of room.† There have been lately many very excellent surgical cases, most of which have been rendered the subjects of clinical lectures, by one or other of the surgeons.

I. LITHOTOMY.—In one case, Mr. White operated for stone, under circumstances deserving of attention. The patient, Joseph Green, 74 years of age, had suffered from stone in the bladder for 7 years. The incisions were made with the greatest facility; but, on seizing the stone, it broke between the blades of the forceps, into a great number of small pieces, apparently composed of the triple phosphates. These rendered the repeated introduction of the forceps necessary; and the smallest pieces were washed out of the bladder by a stream of warm water, thrown in from an injecting syringe, repeated several times. Mr. White then plugged up the opening in the bladder by lint, crammed into it on the end of the finger, so as to prevent the urine passing out, and infiltrating the cellular membrane, if such accident were likely to happen. Towards night, the patient experienced some pain and uneasiness in the region of the bladder, which Mr. White relieved, by removing the lint, and allowing the urine to flow, which it did in a stream, bringing with it some sabulous matter. During the two next days, there was pain above the pubes; but this was relieved by the application of leeches, and attention to the state of the bowels. The febrile derangement was rather of a low kind, and the patient early required support. He is now, near three weeks after the operation, going on well—the urine still flows through the wound, but he is otherwise relieved from his sufferings. The introduction of a piece of lint into the bladder, to act as a syphon, and to prevent union of the cut edges of it, has been often done; but the filling up of the opening, so as to prevent any urine flowing out, deserves further attention.

II. HYDROCELE.—Several cases of hydrocele, of the sacculated kind, formed the subject of a clinical lecture, delivered by Mr. Guthrie. In the first, the patient, J. Moylen, 22 years old, had laboured under the complaint for 6 years, and had been in

---

† We sincerely hope that no selfish interests may interpose between the removal of the hospital to the proposed site near Charing-Cross. The patients, the surgeons, the whole economy of the institution, would profit by the change of situation.—ED.

three different hospitals. It had been injected—the fluid had been let out—and it had been punctured by the trocar, without any fluid being evacuated, which led the surgeon to talk of a further operation, and the patient to make his escape, lest he might leave his testes behind him.* On being admitted into the Westminster Hospital, he said the surgeons called his case a sacculated one. Mr. Guthrie drew off the water, and injected the tunica vaginalis with a solution of sulphate of zinc in water, ʒj. to ℔j. This was retained for nearly 10 minutes, keeping the sac pretty fully distended. The result was, considerable inflammation, which gradually subsided, leaving the testes a little enlarged; which enlargement disappeared under the use of the ung. hydr. c. camphorâ. The man is quite well.

In a second case, the patient (Bedfont) had an enlargement of the scrotum, which had existed for several years. By some surgeons, it was said to be a disease of the testes; by others, of the spermatic cord. The swelling was large, soft and irregular; extending, apparently, along the cord, giving a lobulated feel to the whole; and was, in some parts, of greater firmness than in others, although a fluctuation was perceptible throughout. The testis could be distinguished at the under and back part, more by the pain it gave rise to on pressure, than by its actual presence under the fingers. Mr. Guthrie punctured the swelling with a lancet and probe, and drew off about two drachms of fluid. A second puncture evacuated about 8 drachms; and a third puncture, in another place, nearly as much more. Mr. Guthrie, from these circumstances, and the general lobulated feel of the part, considered the disease to be hydrocele of the tunica vaginalis, and of different sacs attached to the cord. On the Saturday following, he made an incision along the front of the tumour about four inches in length; and, on dividing the tunica vaginalis, about three ounces of fluid escaped; but the large transparent sacs attached to the cord, and overhanging the testes, being still evident, he cut into them with the scissars, and removed portions in every direction, until the whole of the fluid was evacuated. The parts were then brought together with a suture and by sticking plaster. Inflammation soon set in, and the testicle swelled to a greater size than the hydrocele had previously attained. Under the use of cold applications, followed by the ung. hydr. camphorat. it gradually diminished, and the patient was discharged at the end of six weeks, cured.

Mr. Guthrie, in his clinical remarks on these cases, said, that a sacculated hydrocele was comparatively of rare occurrence; he had never seen one so well marked as in the instance of Bedfont, in whom it was rendered manifest by the fluctuation, the peculiar poise which a fluid gives when it rests on the hand, and the soft, yet lobulated, feel the whole had when carefully examined. The three punctures discharged a quantity of fluid, greater than can be obtained from a cysted testicle, in which the fluid is contained in cells, each seldom larger than a full-sized grape, and from which disease it was distinguishable by its greater softness, distinct fluctuation, and the lobulated feel—neither diseases being painful, except when pressure is applied. The cystic testicle was, he said, a disease of the whole body of the testes, incurable, not malignant; yet requiring extirpation, to remove an inconvenience. In the first case, or that of Moylen, he had succeeded in curing the patient, where three different surgeons had failed, by attending (as he believed) to the circumstance of distending the tunica vaginalis fully as much as, if not even a little more than, it had been before the fluid was evacuated; and retaining the injection for some minutes—always for five, and, in doubtful cases, for eight or even ten minutes. If these points were strictly attended to, he believed it signified little of what the injection was composed. He had frequently used cold water alone; and, in two cases, the inflammation rose so high as to terminate in suppuration, rendering an opening into the tunica vaginalis necessary for the evacuation of the matter. Tbe pain felt at the moment of operating, is not a criterion by which we are to judge. Those who suffer the most during the operation, either in the part or in the back, usually suffer the least afterwards,

* This case made a considerable noise some time ago, and gave rise to some badinage in our facetious *weekly* cotemporaries, under the head of "DRY TAPPING."

and *vice versa*—probably from the operator's yielding (in the latter case) to their wishes, and not distending the sac sufficiently; or in drawing off the injection too soon. The idea commonly entertained, that adhesion takes place between the inside of the sac and the testis, is, he said, enormous;—it is a rare occurrence. The first effect of the injection is to excite an increased action of the vessels of the part, followed by an effusion of fluid, which often, in 24 hours, causes the swelling to be as large as before. Sometimes the testis inflames and enlarges very much; but it does not always do so. The action subsiding, the effusion and increased secretion cease;—and the effused fluid is removed by absorption, shewing the disease to have been one of increased secretion, not of diminished absorption.

Hydrocele is apt to return, and to require a second or a third injection, which sometimes does not even then succeed. Recourse should, in such cases, be had to the old operation by incision. Mr. Guthrie said he had been obliged to operate, in this manner, four times this year, in cases from the country, and from abroad, in which injections had failed. In one case of a young noblemen, (whose name we suppress,) who had been three times operated upon, a sort of hour-glass division had taken place in the tunica vaginalis, which, on being laid open, was shewn to arise from an attachment of the tunica vaginalis to the tunica albuginea of the testis, and which it was necessary to divide. It is curious that, after the operation by incision, and what is supposed to the cure by granulation, the cavity of the tunica vaginalis is not always obliterated. He had been obliged to perform the operation in several instances for the second time, on the same side. It was true, a certain period of time had elapsed between the operations, and, in one case, it was the third time the patient had submitted to the operation—but these cases, he said, were unusual.

III. AMPUTATIONS.—Mr. Guthrie also amputated two arms, and made these cases the subject of another clinical lecture. In the first, the arm was removed in a patient greatly debilitated, in consequence of a long-continued scrofulous disease of the wrist, accompanied by sinuses extending up the fore-arm, which was taken off by a double flap operation. On the second day after the amputation, the pulse rose to 96—the stump became swelled and painful, with considerable heat of skin. The bandage and plasters were removed, and a warm poultice was applied, after 20 leeches had abstracted as much blood as they could obtain from the part immediately above the stump. Mr. Guthrie expressed his fears that inflammation of the veins would take place. This apprehension was, however, relieved in the course of the fourth day, by the appearance of its having occurred in the absorbents—a red streak marking its course up to the axilla, where a large abscess formed. This, he said, was a favourable sign, as it rendered the life of the patient secure: whereas, a corresponding extension of inflammation in the veins would have been certainly fatal. The abscess in the axilla required to be largely opened, and several sinuses to be divided, before it healed. The stump, which did not unite by the first intention, took on an unhealthy scrofulous action, but was nearly well when the man was made an out-patient for want of room; his general health being in a good condition.

In the second case, the patient (Howard) had been about the hospital for two years or more, in consequence of an injury to the wrist from the fall of a stone, which tore away the os cuneiform, and injured several other bones of the carpus. The wound had got comparatively well; but being frequently painful, particularly on attempting to use it, and finding he could not get his livelihood, he determined on having it removed;—lest, as he said, "he should be starved to death." The amputation was performed above the wrist by the double flap operation; and it healed by the first intention, with the exception of the small apertures, through which two ligatures came out. From the third day after the operation, the pulse began to rise, and although the patient looked almost a skeleton, it was clear, from the uneasiness in the chest and side, that an insidious attack on the lungs was impending. Twelve ounces of blood were drawn from the arm, with the greatest relief, (the blood shewing the buffy coat,) and being followed up by saline purgatives. The chest was completely relieved. The symptoms, however, returned; and were again and again removed by similar treatment; so that, in five weeks, the patient required to be bled four times. The man is now a labourer, capable of earning his bread. Mr. Guthrie remarked, that 20 years ago this man would, in all probability, have been lost from inflammation of the lungs, with purulent deposits there, or on the cavity of the chest; for few surgeons would have thought of bleeding a patient in the starved state to which this man was reduced. Yet, if this had not been done, and the system of purgation and abstinence strictly enforced, he had no doubt the termination of the case would have been very different.

---

